# IgG subclasses in cryoglobulins: link to composition and clinical manifestations

**DOI:** 10.1186/s13075-020-02364-z

**Published:** 2020-11-12

**Authors:** Marie N. Kolopp-Sarda, Pedro Ming Azevedo, Pierre Miossec

**Affiliations:** 1 Immunogenomics and Inflammation research Unit EA 4130, University of Lyon, Lyon, France; 2 Immunology Laboratory, Hospices Civils de Lyon, Lyon, France; 3 Department of Immunology and Rheumatology, Clinical Immunology Unit, Hospices Civils de Lyon, Lyon, France

**Keywords:** Cryoglobulins, IgG subclasses, Vasculitis, Complement, Rheumatoid factor

## Abstract

**Background:**

Cryoglobulins (CG) are immunoglobulins which precipitate at low temperature. The analysis of IgG subclass composition of CG is poorly reported. The aim of this study was to determine the subclasses of IgG-containing type I and mixed type II and III CG in relation to clinical manifestations.

**Methods:**

Out of a previous series of 1675 patients, inclusion criteria were a cryoprecipitate > 1 mL and a total IgG > 300 mg/L. IgG subclasses were quantified by immunoturbidimetry, rheumatoid factor (RF), and C4 by immunonephelometry. Clinical parameters were collected from hospital charts.

**Results:**

CG samples from 86 patients were included, 10 type I CG and 76 mixed CG. Type I CG subclasses were IgG1 (6/10) and IgG2/IgG3 (4/10), never IgG4. IgG subclass in type II vs. III CG were 73.3 ± 15.2% vs. 52.5 ± 20.7% for IgG1 (*p* < 0.0001), 15.4 ± 8.2% vs. 25.9 ± 14% for IgG2 (*p* < 0.0001), 8.4 ± 12.4 vs. 21.2 ± 14% for IgG3 (*p* < 0.0001), and 3 ± 5.2% vs. 0.5 ± 1.2 for IgG4 (*p* < 0.0001). In mixed CG, the higher proportion of IgG4 was associated with RF positive CG (*p* = 0.01) and low C4 (*p* = 0.01). In type I CG, IgG1 were associated with severe vasculitis manifestations, IgG2/IgG3 with cutaneous or renal manifestations. In mixed CG, IgG2 was the only subclass associated with CG manifestations, with a higher concentration in asymptomatic (162.6 ± 29.5 mg/L) vs. symptomatic patients with cutaneous (103 ± 17.8 mg/L, *p* = 0.04) and neurological (108 ± 24 mg/L, *p* = 0.04) manifestations.

**Conclusion:**

In type I IgG CG, IgG1 was the main CG subclass associated with CG vasculitis. In mixed CG, low IgG2 concentration was linked to CG cutaneous and neurological manifestations.

## Introduction

Cryoglobulins (CG) are immunoglobulins (Ig) which precipitate at low temperature. They are classified as type I CG, composed of a monoclonal Ig (IgM or IgG), type II CG composed of a monoclonal and polyclonal Ig, and type III CG composed of polyclonal Ig. Type II and type III CG are called mixed CG, with monoclonal or polyclonal IgM with rheumatoid factor (RF) activity (IgM anti-IgG) [1].

IgG is the most important Ig class of the humoral immune response, with 4 subclasses with structural differences of the heavy chain, with distinct physicochemical, biological, and immunological properties. The IgG effector functions, such as immune complex formation, complement binding, and activation, depend on the Fc fragment [2]. Larger immune complexes are formed with IgG1 and IgG3, which efficiently bind C1q and other complement fractions to activate the classical pathway. Due to its spatial structure, CH2 glycosylation, and difficult accessibility of CH domains, IgG4 cannot form immune complexes and activate complement by the classical pathway [3]. Immune complex formation and complement activation are involved in CG pathogenesis [4]. Different IgG subclasses are involved in the pathogenesis of immune complexes, in particular in autoimmune renal diseases [5, 6]. This topic has been rarely reported in the literature for cryoglobulinemia.

Few studies had reported on the determination of IgG subclasses in human CG [7–13], often with small size series. In mouse models, cryoprecipitating IgG CG were IgG3, with cutaneous and renal vasculitis equivalent to CG vasculitis in humans [14–17]. In mixed human CG, all IgG subclasses were found in the cryoprecipitate, with an enrichment in IgG3, possibly because of its property to self-aggregate or to have RF activity [18–20]. No clear relation between the IgG subclass composition of CG and CG vasculitis was described in humans [19–21].

The purpose of the present study was to focus on the IgG subclasses and their associated clinical manifestations, in type I and mixed CG from a previous series of 1675 characterised CG [22].

## Material and methods

### Population and data collection

The present retrospective study was conducted at the University Hospitals of Lyon, France. From 1675 patients with positive detection of CG tested in a previous study [22], this study included samples for whom a cryoprecipitate was kept in the cryoglobulin bank. Inclusion criteria for subclass identification were a cryoprecipitate volume > 1 mL and a total IgG concentration > 300 mg/L. Cryoprecipitate volume needed to determine subclasses was at least 300 μL. These inclusion criteria related to volume and IgG concentration in the cryoprecipitate may have induced some patient selection bias, in particular for the relationships between subclasses and clinical signs.

Clinical data possibly associated with CG or with underlying conditions were collected retrospectively from the hospital unified electronic medical records. Symptoms were classified as cutaneous (Raynaud phenomenon/acrocyanosis, livedo, purpura, ulcers), neurological (peripheral neuropathy), renal (glomerulonephritis, haematuria, proteinuria), and rheumatologic (arthralgia, arthritis, myalgia).

The protocol was approved by the Ethics Committee of the Hospitals of Lyon for the protection of the people participating in biomedical research under the number AC-2016-2729.

### CG characterisation

All CG were detected and analysed as previously described [22]. Total IgG concentration in the cryoprecipitate was measured by immunonephelometry (BN ProSpec®, Siemens Healthcare, St Denis, France), and the four IgG subclasses were assessed by immunoturbidimetry (Optilite®, The Binding Site, St Egrève, France). The sensitivity limit inherent to this immunoturbidimetric method (150 mg/L for IgG1, 20 mg/L for IgG2, 10 mg/L for IgG3, 5 mg/L for IgG4) prevented assessment of interpretable subclass repartition when the total IgG concentration in the cryoprecipitate is less than 300 mg/L. The proportion of IgG subclass was calculated by dividing the concentration of each IgG subclass by the concentration of total IgG in each sample. Mean proportions of IgG subclasses in normal adult serum are IgG1 60%, IgG2 32%, IgG3 4%, and IgG4 4% [2].

RF activity of CG (IgM anti-IgG) was measured in the cryoprecipitate by immunonephelometry (normal < 10 IU/mL, BN ProSpec®, Siemens). Complement fraction C4 concentration was measured with BN ProSpec® (Siemens) in serums collected simultaneously with the CG testing sample. C4 normal range in the laboratory is 0.14–0.32 g/L.

### Statistical analysis

For continuous variables, the results were expressed as mean ± SEM or median (range) when the distribution was not normal. Distribution normality was tested using the D’Agostino Pearson omnibus normality test. The Kruskal-Wallis test was used for the variance analysis and the Mann-Whitney test for the comparison of quantitative variables. The Student *t* test and Wilcoxon test were used for the comparison of quantitative parameters. Chi-squared test and Fisher’s exact test were used to analyse qualitative differences. *P* < 0.05 was considered statistically significant. Calculations were performed with GraphPad Prism version 5.01 software (La Jolla, CA, USA).

## Results

### Population and IgG subclass in the cryoprecipitates

From the 1675 patients, 86 were included in this study based on inclusion criteria (cryoprecipitate > 1 mL and cryoprecipitate IgG concentration > 300 mg/L). There was no significant difference for demographical data of this cohort compared to the initial larger cohort [22]. Sex ratio in the larger cohort was 1.55 (1018F/657M) compared to 1.39 in the subclass series (50F/36M, *p* = 0.57). The mean age in the larger cohort was 54.0 ± 17.4 years compared to 58.7 ± 15.5 years in the subclass series (*p* = 0.25). All type I IgG CG were in the two cohorts. For the mixed CG, 40.8% were secondary to infectious diseases (mostly HCV) in this cohort vs. 49.5% in the larger cohort (*p* = 0.18); 28.9% were secondary to auto-immune diseases in this cohort vs. 25.4% in the larger cohort (*p* = 0.49).

Among the 86 patients included, there were 10 patients with type I IgG CG (6 IgG kappa and 4 IgG lambda), and 50 patients with type II CG (including 41 RF-positive CG) and 26 patients with type III CG (including 8 RF-positive CG) (supplementary Fig. S1). Out of these 86 patients, 100 samples were analysed (12 samples from 10 patients with type I CG, 62 samples from 50 patients with type II CG, and 26 samples from 26 patients with type III CG), because some patients had several CG detections and the cryoprecipitates have been banked. These replicates were used to test the assay reproducibility and stability of CG composition. The comparison of paired values did not show any difference for IgG1 (*p* = 0.7), IgG2 (*p* = 0.7), IgG3 (*p* = 0.2), and IgG4 (*p* = 0.6).

Among the 50 patients with type II CG, 46 CG were monoclonal IgM (IgM kappa and IgM lambda) associated with polyclonal IgG, and 4 CG were monoclonal IgG (3 IgG kappa and 1 IgG lambda) associated with polyclonal Ig (IgG, IgA, or IgM). All 26 type III CG samples were polyclonal IgG by definition.

For IgG type I and mixed CG, IgG1 was the most represented subclass (67.4 ± 19.2%) as in the serum (60%), then IgG2 (18.4 ± 11.1% vs. 32% in the serum), IgG3 (12 ± 14% vs. 4% in the serum), and IgG4 (2.2 ± 4.6% vs. 4% in the serum). This repartition was significantly different from the normal serum IgG subclass repartition with less IgG2 and more IgG3 (*p* = 0.03).

The proportion of CG types in the four subclasses showed that IgG1 were found in all types of CG (*p* = 0.25), IgG2 were more frequent in type III CG vs. type II and type I CG (*p* < 0.001), IgG3 were more frequent in type I CG vs. type III and type II CG (*p* < 0.001), and IgG4 were more frequent in type II CG vs. type III CG and never in type I CG (*p* < 0.0001).

### IgG subclasses in type I cryoglobulins

Out of the 10 patients with type I IgG CG, IgG subclasses were 5 IgG1 (2 IgG1 kappa and 3 IgG1 lambda), 2 IgG2 (1 IgG2 lambda and 1 IgG2 kappa for 84% associated for 16% with an IgG3), and 2 IgG3 (1 IgG3 kappa and 1 IgG3 lambda for 82.2% associated for 17.8% with an IgG2). One patient had a biclonal type I CG, with the same proportion of IgG1 and IgG3. No type I CG contained IgG4 (Table 1).

**Table 1:**
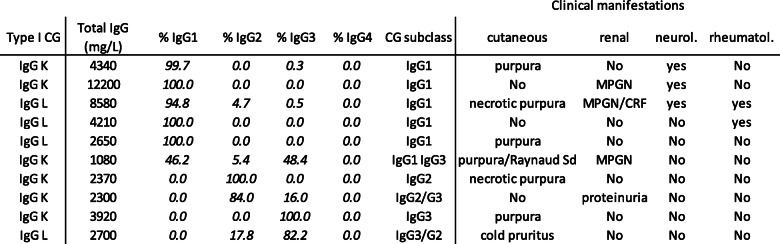
IgG subclass composition of type I cryoglobulins

*MPGN* membranoproliferative glomerulonephritis, *CRF* chronic renal failure

### IgG subclasses in mixed cryoglobulins

**Fig. 1:**
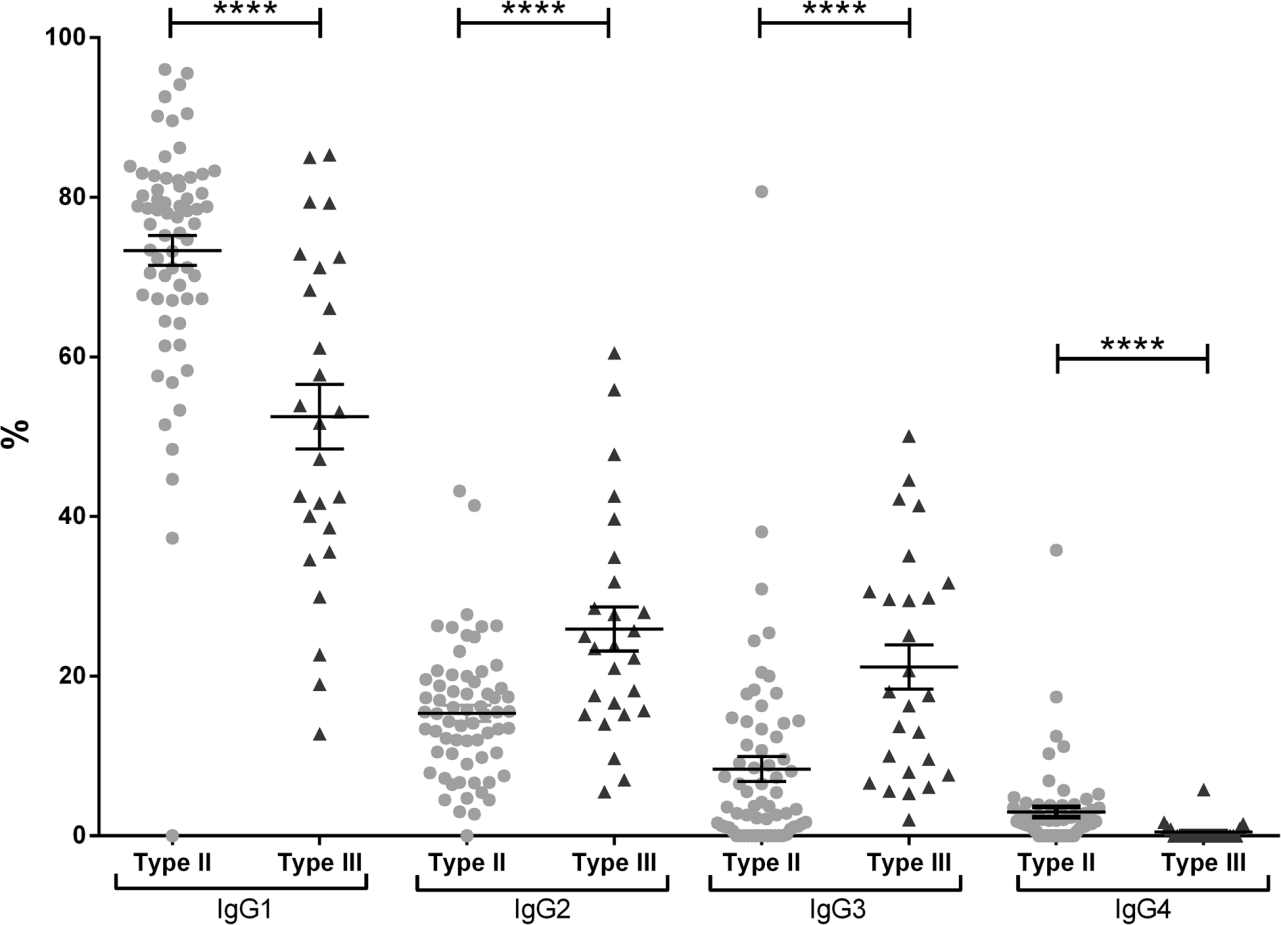
Subclass proportion in mixed cryoglobulins. Express as the percentage of total IgG in the cryoprecipitate (mean ± SEM); type II CG: light grey points; type III C: dark grey triangles; ****p < 0.0001

The IgG subclass proportions in type II and III CG were for IgG1, 73.3 ± 15.2% vs. 52.5 ± 20.7% (*p* < 0.0001), for IgG2 15.4 ± 8.2% vs. 25.9 ± 14% (*p* < 0.0001), for IgG3 8.4 ± 12.4 vs. 21.2 ± 14% (*p* < 0.0001), and for IgG4 3 ± 5.2% vs. 0.5 ± 1.2 (*p* < 0.0001) (Fig. 1).

There was no significant difference between total IgG concentration in the cryoprecipitate of type II and type III CG (respectively 761 ± 113 mg/L vs. 663 ± 174 mg/L, *p* = 0.8), but IgG1 concentration was higher in type II CG than in type III CG (586 ± 94.7 vs. 416 ± 154 mg/L, *p* = 0.02), and IgG2 and IgG3 concentrations were higher in type III CG than in type II CG (IgG2, 105 ± 17.7 vs. 130 ± 13.9 mg/L, *p* = 0.004; IgG3, 49.8 ± 8.4 vs. 105 ± 13 mg/L, *p* < 0.0001); IgG4 concentration was higher in type II CG than in type III CG (20.8 ± 4.5 vs. 12 ± 10.9 mg/L, *p* < 0.0001) (supp Fig. S2).

### IgG subclass and RF activity

**Fig. 2:**
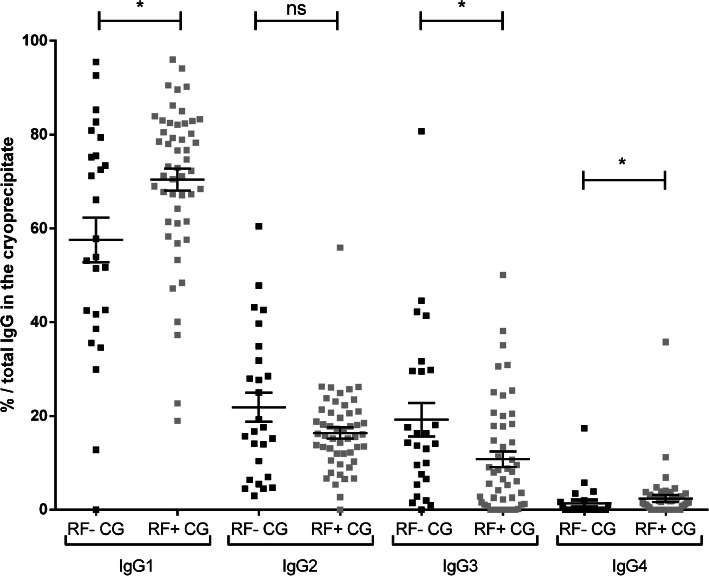
Comparison of RF activity in function of subclass composition of type II and type III cryoglobulins. Express as subclass proportion (the percentage of total IgG in the cryoprecipitate); RF− CG: black square; RF+ CG: grey square; *p = 0.02, *ns* non-significant by Mann-Whitney test

For the mixed CG, there were 26 RF-negative CG (9 type II, 17 type III) and 50 RF-positive CG (41 type II, 9 type III). IgG subclass proportions in RF-negative CG compared to RF-positive CG were for IgG1 57.6 ± 4.8% vs. 70.4 ± 2.4% (*p* = 0.02), for IgG2 21.9 ± 3.1% vs. 16.4 ± 1.2% (*p* = 0.31), for IgG3 19.2 ± 3.6% vs. 10.8 ± 1.7% (*p* = 0.02), and for IgG4 1.4 ± 0.7% vs. 2.4 ± 0.7% (*p* = 0.02) (Fig. 2). Comparison of subclass concentrations in RF-negative CG vs. RF-positive CG showed no differences for IgG1 (428 ± 154 vs. 579 ± 95 mg/L, *p* = 0.09) and IgG2 (114 ± 16.8 vs. 114 ± 17.2 mg/L, *p* = 0.52) and a significant difference for IgG3 (91.8 ± 14.8 vs. 56.5 ± 8.4 mg/L, *p* = 0.04) and IgG4 (15.3 ± 11 vs. 19.1 ± 4.5 mg/L, *p* = 0.01) (supp Fig. S3).

### IgG subclass and decrease of complement C4

**Fig. 3:**
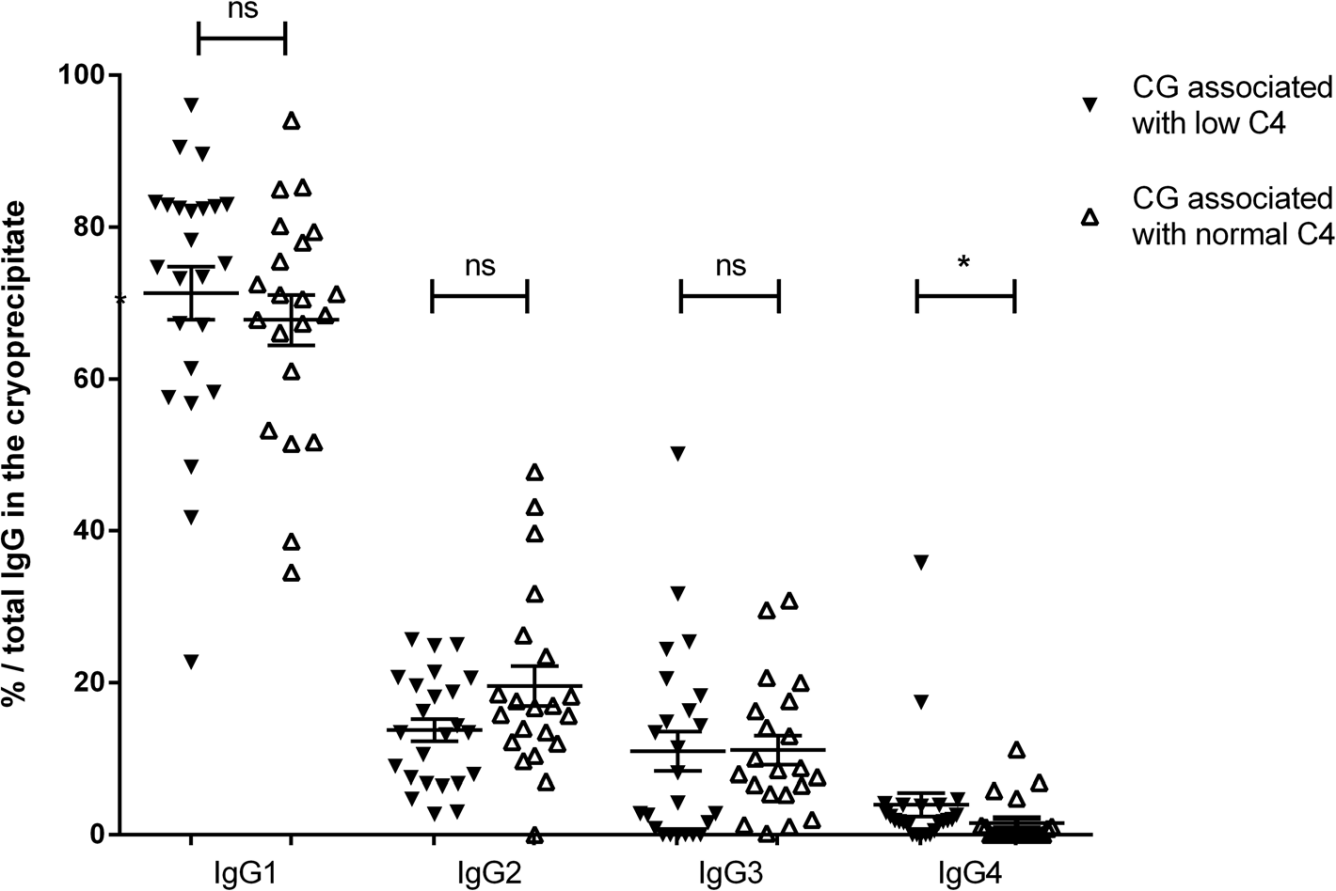
Subclass proportion of type II and type III CG for patients with low complement C4 fraction (C4 ≤ 0.10 g/L) and normal C4 fraction (> 0.10 g/L) in the serum. Express as the percentage of total IgG in the cryoprecipitate (mean ± SEM); Low C4: black triangle; normal C4: white triangle; *p = 0.01, *ns* non-significant, by Mann-Whitney test

For the mixed CG, 45 samples had a complement exploration at the time of CG detection: 24/45 (53.3%) had a low C4 concentration (≤ 0.10 g/L) and 21/45 (46.7%) a normal C4 concentration (> 0.10 g/L). Comparison of subclass constitution of mixed CG associated with low or normal C4 showed no difference for the proportion of IgG1 (71.3 ± 3.5 vs. 67.8 ± 3.3%, *p* = 0.3), IgG2 (13.8 ± 1.5 vs. 19.6 ± 2.6%, *p* = 0.15), and IgG3 (11 ± 2.6 vs. 11.1 ± 1.9%, *p* = 0.43), but IgG4 proportion was higher with low (4 ± 1.6%) vs. normal C4 (1.6 ± 0.7%, *p* = 0.01) (Fig. 3). A significant correlation was found between IgG1 and IgG4 in RF-positive CG (*p* = 0.04, supp Fig. S4), but not with IgG2 and IgG3 (*p* = 0.25 and *p* = 0.10, respectively).

### Subclass composition and clinical manifestations of cryoglobulins

**Fig. 4:**
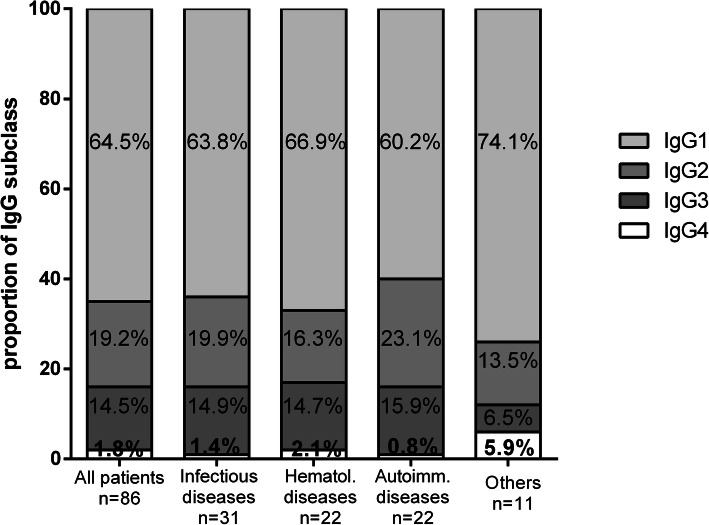
Proportion of IgG subclass in CG according to underlying diseases

Patients were divided into four groups depending on the underlying diseases: 31 infectious diseases (26 HCV infections, 5 non-chronic infections), 22 haematological diseases (8 monoclonal gammopathies of undetermined significance, 3 multiple myeloma, 8 lymphoma, 1 leukaemia, 2 Waldenström disease), 22 autoimmune diseases (7 Sjogren’s syndrome (SS), 2 systemic lupus erythematosus (SLE), 2 association of SLE and SS, 11 unclassified connective diseases with anti-nuclear antibodies), and 11 others (9 idiopathic CG, 1 cirrhosis, 1 metastatic gastric cancer). Analysis of IgG subclass variances between these 4 groups of underlying diseases showed a lower proportion of IgG2 in CG secondary to haematological diseases (16.3 ± 5.4%) than in infectious (19.9 ± 2%, *p* = 0.02) and autoimmune diseases (23.1 ± 3.4%, *p* = 0.004, Fig. 4).

For the 86 patients, 64 (74.4%) presented clinical manifestations related to cryoglobulinemia. Clinical manifestations were in order of frequency: cutaneous (38/64, 59.4%), neurological (29/64, 45.3%), renal (20/64, 32.2%), rheumatological (18/64, 28.1%), and digestive (10/64, 15.6%) (Table 2).

**Table 2:** Clinical manifestations of cryoglobulinaemic vasculitis in all patients

Patients	Total	Type I CG	Mixed	Type II CG	Type III CG	*P*
	*n* = 86	*n* = 10	*n* = 76	*n* = 50	*n* = 26	
Asymptomatic patients, *n* (%)	22 (25.6)	0 (0)	22 (28.9)	9 (18)	13 (50)	0.06^§^/0.007^§§^
Symptomatic patients, *n* (%)	64 (74.4)	10 (100)	54 (71.1)	41 (82)	13 (50)	
Clinical manifestations:						
Cutaneous	38 (59.4)	7 (70)	31 (40.8)	24 (48)	7 (26.9)	0.10^§^/0.09^§§^
Neurological	29 (45.3)	4 (40)	25 (32.9)	19 (38)	6 (23.1)	0.73^§^/0.21^§§^
Renal	20 (31.2)	4 (40)	16 (21)	13 (26)	3 (11.5)	0.23^§^/0.23^§§^
Rheumatological	18 (28.1)	2 (20)	16 (21)	12 (24)	4 (15.4)	0.99^§^/0.55^§§^
Digestive	10 (15.6)	2 (20)	8 (10.5)	3 (6)	5 (19.2)	0.33^§^/0.11^§§^

Cutaneous signs: Raynaud phenomenon/acrocyanosis, livedo, purpura, ulcers; Neurological signs: peripheral neuropathy; Renal signs: glomerulonephritis, haematuria, proteinuria; Rheumatological signs: arthralgia, arthritis, myalgia; Digestive signs: intestinal pain

*n* number of patients, *%* percentage/number of patients of each column, *Mixed column* type II and type III CG

^*§*^Comparison between type I and mixed CG

^§§^Comparison between type II and type III CG

### IgG subclasses and clinical manifestations of type I CG

Patients with type I IgG CG were all symptomatic. Among the 6 patients with IgG1, 4 presented cutaneous signs associated with neurological (3/4), articular (1/4), and/or renal (3/4) signs, and 2 had no cutaneous signs but neurological, renal, and/or articular manifestations. The 4 patients with IgG2 and/or IgG3 had cutaneous manifestations only (3/4), and one with renal manifestation (proteinuria) (Table 1).

### IgG subclasses and clinical manifestations of mixed CG

Among the 76 patients with mixed CG, 22/76 (28.9%) were asymptomatic patients (9 with type II CG and 13 with type III CG), and 54/76 (71.1%) were symptomatic. Cutaneous signs were the most frequent manifestation, found in 48% (24/50) of type II CG and 26.9% (7/26) of type III CG, neurological signs were found in 38% (19/50) of type II CG and 23.1% (6/26) of type III CG, renal signs were found in 26% (13/50) and 11.5% (3/26), and articular signs were found in 24% (12/50) and 15.4% (4/26). More digestive manifestation was found in type III (19.2%, 5/26) than in type II CG (6%, 3/50) (Table 2).

Comparison of subclass constitution of mixed CG in asymptomatic and symptomatic patients showed no significant difference for the proportion of IgG1 (58.7 ± 4.9% vs. 68.9 ± 2.5%, *p* = 0.08), IgG3 (16.3 ± 3 vs. 12.6 ± 2%, *p* = 0.19) and IgG4 (0.95 ± 0.3 vs. 2.6 ± 0.7%, *p* = 0.08); but the IgG2 proportion was higher in asymptomatic patients (24 ± 3.3%) than in symptomatic patients (15.9 ± 1.2%, *p* = 0.03). No difference was found between asymptomatic and symptomatic patients for the concentration of IgG1 (660 ± 216 vs. 480 ± 75 mg/L, *p* = 0.5), IgG3 (80.9 ± 13.9 vs. 63.7 ± 9.2 mg/L, *p* = 0.2), and IgG4 (21.3 ± 13.3 vs. 16.7 ± 4 mg/L, *p* = 0.18), but IgG2 was higher in asymptomatic (162.6 ± 29.5 mg/L) vs. symptomatic patients (94.3 ± 12.3 mg/L, *p* = 0.02). This difference of IgG2 concentration was associated with cutaneous (103 ± 17.8 mg/L, *p* = 0.04) and neurological manifestations (108 ± 24 mg/L, *p* = 0.04) compared to asymptomatic patients (162.6 ± 29.5 mg/L). No difference in IgG2 concentration and proportion were found for renal and rheumatological manifestations in symptomatic compared with asymptomatic patients.

## Discussion

Type I IgG CG were mostly IgG1, in relation with cutaneous, renal, and neurologic manifestations. Some IgG2 and IgG3 type I CG were observed, associated with cutaneous or renal manifestations. In mixed CG, IgG1 were more frequent in type II CG, associated with RF-positive CG, and IgG2 and IgG3 in type III CG. A higher proportion of IgG4 was associated with RF-positive CG and a low level of C4. IgG2 concentration was lower in the mixed CG of symptomatic patients.

For type I IgG CG, few studies have reported on the determination of IgG subclasses and their relation to the associated clinical manifestations [7–10, 13]. In this study, IgG1 was the most common cryoprecipitating type I IgG and no monoclonal IgG4 was found, as reported previously [7, 10]. The distribution of monoclonal IgG subclasses was different between cryoprecipitating IgG and normal IgG, with more IgG2 and IgG3 precipitating in the cold and rarely, if any, IgG4 [23, 24]. Monoclonal IgG1 and IgG3 with their high capacity to aggregate are good candidates for cryoprecipitation. In contrast, cryoprecipitation of IgG4 seems more difficult due to its peculiar conformation and lower capacity to self-aggregate and to form large size complexes [2, 3, 25].

An association of two subclasses in type I CG was reported in this series: monoclonal IgG1 and IgG3 in the same proportion for one patient, and IgG2/IgG3 association for two patients. The association of more than one IgG subclass within type I CG and normal monoclonal IgG could result from one plasma cell clone producing several gamma heavy chains associated with the same light chain [11] or, that more than one pathologic plasma cell clones were involved in monoclonal gammopathies [24]. Another hypothesis for the association with monoclonal IgG3 was the RF activity of IgG3 recognising IgG Fc (IgG3 RF anti-IgG) leading to the formation of larger size complexes when temperature decreases [12, 20].

Regarding the link to clinical manifestations in this study of type I CG, IgG1 was associated with multiple vasculitis manifestations (cutaneous, neurological, rheumatological, and/or renal), and IgG2 and IgG3 were associated with only one type of manifestation (cutaneous or renal). Only few clinical cases were previously reported concerning patients with type I IgG CG [8, 9, 13], not allowing a conclusion on a relationship between IgG subclass and clinical manifestations. Cutaneous manifestations were the main clinical signs reported in this series; nephrotoxicity was rarely observed, only in patients with IgG1 as reported in another case [13]. In the autoimmune mouse MRL model and in humans, IgG3 CG was described as nephrotoxic [9, 14–17, 26, 27]. Mouse IgG1 was reported as protecting against renal disease in a mouse model of cryoglobulinemia, unlike IgG3 [28]. Mouse IgG3 can self-associate to form large immune complexes which could precipitate in glomeruli capillaries [28]. These mouse models cannot be transferred to humans, because mouse IgG1 resembles human IgG4, not involved in type I CG pathophysiology. The involvement of one specific IgG subclass in renal manifestations in humans is not clear because renal manifestations appear several years after CG diagnosis [29]. This delay could explain differences in the reported results and justify a long follow-up study of patients.

Regarding mixed CG, IgG1 was the main subclass especially in type II CG, IgG2, and IgG3 were overrepresented in type III CG, and IgG4 was less represented. In studies reporting on mixed CG, all IgG subclasses were found with similar distribution as reported here, especially in RF-positive CG [16, 27, 28].

The significant association of IgG2 and IgG3 in mixed CG observed in this study may be linked to RF activity of IgG3 (IgG3 anti-IgG2), responsible for immune complex formation at low temperature [12, 14, 20, 26]. In this series, IgG3 were found in higher proportion and concentration in RF-negative mixed CG. This higher representation of IgG3 was not in relation with their association with RF IgM as previously reported [18, 30, 31], but could be due to their greater ability to form spontaneous aggregates and to cryoprecipitate.

There was a trend for a higher concentration of total IgG in mixed CG in asymptomatic patients, compared to symptomatic ones that could be explained by an earlier diagnosis of CG in symptomatic patients. However, in patients with cutaneous and neurological manifestations, IgG2 was found less represented in mixed CG than in asymptomatic patients. Regarding IgG3, no direct relationship to a clinical manifestation was found herein. However, in the literature in the mouse MRL model [14, 15, 17] and humans [9, 16, 17], IgG3 has been linked with renal and cutaneous manifestations of CG. In another model of human mixed IgM-IgG CG transfer in Balbc mice, the crucial role of in situ deposits of CG complexes has been linked to the development of CG glomerulonephritis, independently of RF activity or IgG subclass composition [21]. Clinical manifestation of human mixed CG did not clearly appear to be related to their IgG subclass composition.

Surprisingly, in mixed CG, higher proportion and concentration of IgG4 were associated with RF-positive CG and low C4 concentration, in favour of complement system activation. IgG4 subclass cannot activate complement classical pathway, unlike IgG1, because mutations in the γ4 CH2 domain inhibit the binding of C1q [2]. In this study, IgG4 concentration was significantly associated with that of IgG1 in CG immune complexes, which favour complement activation.

## Conclusion

In type I IgG CG, IgG1 was the main CG subclass associated with severe CG clinical manifestations. IgG1 involvement will be confirmed by a long-term follow-up of patients with cryoglobulinaemic vasculitis. In mixed CG, the four subclasses were found involved in CG complexes and only IgG2 was linked to cutaneous and neurological manifestations. The role of each subclass in mixed CG could be clarified in a follow-up study, to define mechanisms of precipitation and their involvement in vasculitis pathophysiology.

## Supplementary Information

**Additional file 1 : Figure S1.** Study flow chart of patient inclusion within the study period (2010–2016).

**Additional file 2 : Figure S2.** Subclass and total IgG concentration in mixed CG. Subclass concentration is express as mg/L (mean ± SEM); type II CG: light grey bars; type III CG: dark grey bars; * *p* = 0.03, ** *p* = 0.001,**** *p* < 0.0001, ns: non-significant compared by Mann Whitney test.

**Additional file 3 : Figure S3.** Subclass concentration in RF-positive and RF-negative mixed CG (mg/L, mean ± SEM). RF- CG: black bar; RF+ CG: grey bar; * *p*=0.02, ** *p*=0.01; ns: non-significant by Mann Whitney test.

**Additional file 4 : Figure S4.** Correlation between IgG1 and IgG4 in mixed RF-positive CG. This correlation explain the complement activation in CG with IgG4.

## Data Availability

Not applicable

## Abbreviations

CG: Cryoglobulin(s)
Ig: Immunoglobulin
RF: Rheumatoid factor
