# Supplementary figures and images for: IgG subclasses in cryoglobulins: link to composition and clinical manifestations
Source: Arthritis Res Ther. 2020 Nov 12;22:267. doi: 10.1186/s13075-020-02364-z (PMC7659205; doi:10.1186/s13075-020-02364-z)

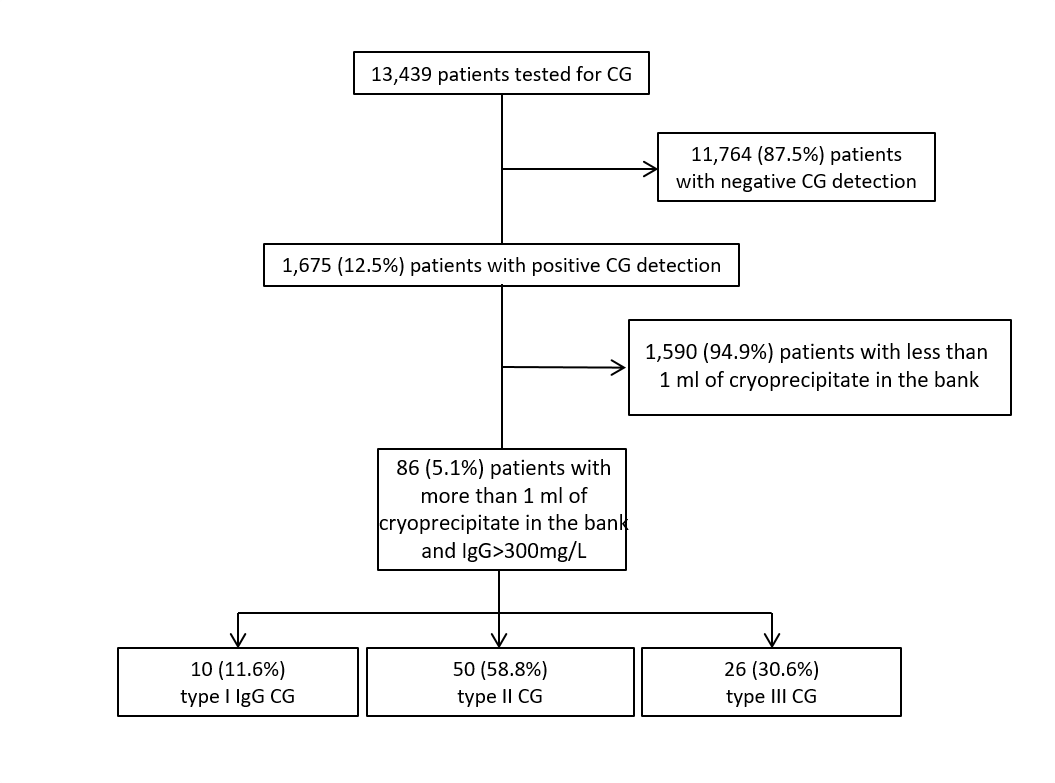

Supplement: Supplementary file 1 — Additional file 1 : Figure S1. Study flow chart of patient inclusion within the study period (2010–2016). [file 13075_2020_2364_MOESM1_ESM.tif]
